# Expression Analysis of Taste Signal Transduction Molecules in the Fungiform and Circumvallate Papillae of the Rhesus Macaque, *Macaca mulatta*

**DOI:** 10.1371/journal.pone.0045426

**Published:** 2012-09-21

**Authors:** Yoshiro Ishimaru, Miki Abe, Tomiko Asakura, Hiroo Imai, Keiko Abe

**Affiliations:** 1 Department of Applied Biological Chemistry, Graduate School of Agricultural and Life Sciences, The University of Tokyo, Bunkyo-ku, Tokyo, Japan; 2 Primate Research Institute, Kyoto University, Inuyama, Aichi, Japan; Duke University, United States of America

## Abstract

The molecular mechanisms of the mammalian gustatory system have been examined in many studies using rodents as model organisms. In this study, we examined the mRNA expression of molecules involved in taste signal transduction in the fungiform papillae (FuP) and circumvallate papillae (CvP) of the rhesus macaque, *Macaca mulatta*, using *in situ* hybridization. *TAS1R1*, *TAS1R2, TAS2Rs, and PKD1L3* were exclusively expressed in different subsets of taste receptor cells (TRCs) in the FuP and CvP. This finding suggests that TRCs sensing different basic taste modalities are mutually segregated in macaque taste buds. Individual *TAS2Rs* exhibited a variety of expression patterns in terms of the apparent level of expression and the number of TRCs expressing these genes, as in the case of human *TAS2Rs*. *GNAT3*, but not *GNA14*, was expressed in TRCs of FuP, whereas *GNA14* was expressed in a small population of TRCs of CvP, which were distinct from *GNAT3*- or *TAS1R2*-positive TRCs. These results demonstrate similarities and differences between primates and rodents in the expression profiles of genes involved in taste signal transduction.

## Introduction

The five basic taste modalities, namely, sweet, bitter, umami (savory), sour, and salty, are detected by taste receptors that are localized at the apical ends of taste receptor cells (TRCs) that form taste buds [1], [2], [3]. Previous studies, mainly in rodents, have demonstrated that sweet, bitter, and umami tastes are mediated by two families of G protein-coupled receptors: T1rs and T2rs [1], [3]. T1r1 and T1r2 form heteromers with T1r3 to function as umami and sweet taste receptors, respectively [4], [5], [6]. The *Tas2rs*, which encode bitter taste receptors, comprise approximately 30 members in mammals [7], [8], [9]. Acting as downstream signal transduction molecules, two G protein α subunits, *Gnat3* (which encodes gustducin) and *Gna14*, phospholipase C-β2 (*Plcb2*), and transient receptor potential melastatin-5 (*Trpm5*) are expressed in subsets of TRCs and play important roles in taste signal transduction [10], [11], [12], [13], [14]. Polycystic kidney disease 1-like 3 (*Pkd1l3*) and polycystic kidney disease 2-like 1 (*Pkd2l1*) are expressed in sour-sensing TRCs [15], [16], [17], [18], [19], [20]. Expression analysis demonstrated that certain genes involved in taste signal transduction exhibited different expression patterns between the fungiform papillae (FuP) and the circumvallate papillae (CvP), which are located on the anterior and posterior regions of the tongue, respectively; *Tas1r1* was expressed primarily in the FuP, whereas *Tas1r2*, *Tas2rs*, *Pkd1l3*, and *Gna14* were expressed primarily in the CvP [7], [10], [13], [17], [19], [21]. In contrast, *Tas1r3*, *Pkd2l1*, *Gnat3*, *Plcb2*, and *Trpm5* were expressed in both the FuP and the CvP [4], [14], [17], [19], [22].

The expression profiles of genes involved in taste signal transduction have been partially uncovered in primates, including humans [23], [24], [25], [26], [27]. *In situ* hybridization (ISH) demonstrated that human *TAS2Rs* were expressed in heterogeneous populations of TRCs [23], whereas the expression of multiple *Tas2rs* occurred in the same subset of TRCs in mice [7]. On the other hand, Matsunami and colleagues demonstrated that each *Tas2r* was expressed in a much smaller number of TRCs than *Gnat3* in mice [9]. The tissue distribution of expression of genes involved in taste signal transduction, including *TAS1Rs*, *TAS2Rs*, *PKDs*, and *TRPM5*, was examined in the CvP of the cynomolgus macaque, *Macaca fascicularis* [24], but the co-expression relationships among these genes largely remain to be elucidated. Moreover, the tissue distribution of expression of the majority of genes in the FuP has not been examined by ISH, except for *PKD1L3* and *TRPM5* [25].

In this study, we examined the mRNA expression of genes involved in taste signal transduction in more detail in the FuP and CvP of the rhesus macaque, *Macaca mulatta*, by ISH. We compared the gene expression profiles in the FuP and CvP and examined the co-expression relationships among various genes. We found both similarities and differences between macaques and rodents. This study may provide new insights into the molecular mechanisms underlying taste sensation in primates, including humans.

**Figure 1:**
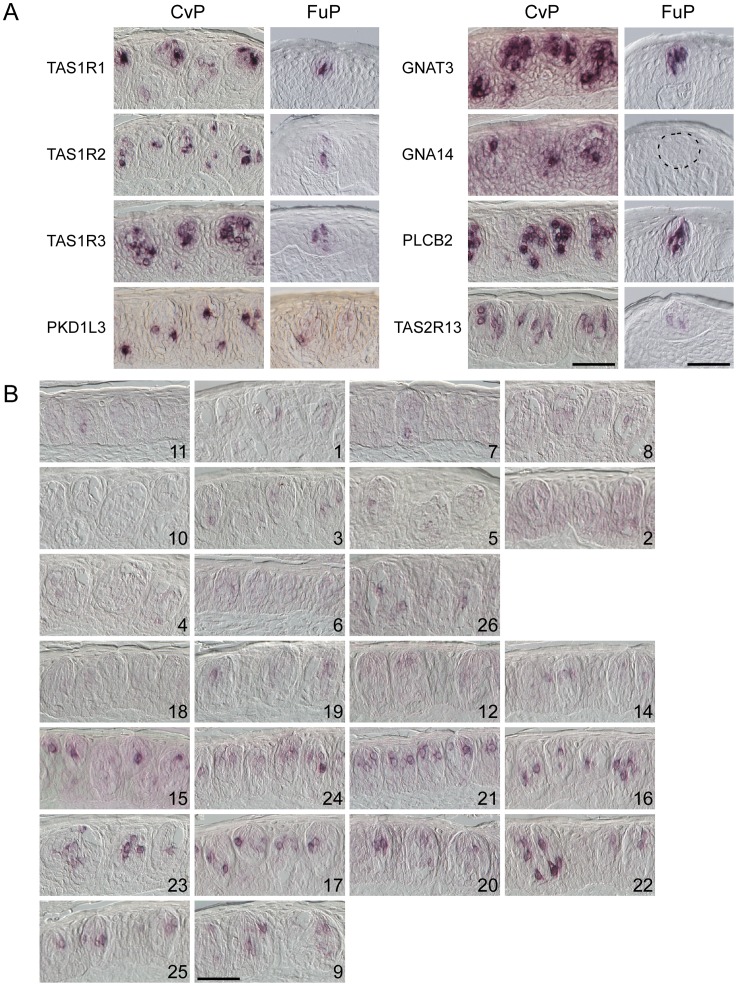
The mRNA expression of genes encoding taste receptors and signal transduction molecules in the fungiform and circumvallate papillae of the rhesus macaque. (A) *In situ* hybridization revealed that three *TAS1Rs*, *TAS2R13*, *PKD1L3*, *GNAT3*, *GNA14*, and *PLCB2* were robustly expressed in subsets of the TRCs in the CvP. These genes, except for *GNA14*, were also expressed in subsets of the TRCs in the FuP. n≥2 (numbers of sections ≥4) for *TAS1R1*, *TAS1R2*, *TAS1R3*, *PKD1L3*, *GNAT3*, and *TAS2R13* in CvP, n = 1 (numbers of sections ≥2) for *GNA14* and *PLCB2* in CvP, n≥2 (numbers of sections ≥20) for *TAS1R1*, *TAS1R2*, *TAS1R3*, *PKD1L3*, and *GNA14* in FuP, n = 1 (numbers of sections ≥10) for *GNAT3*, *PLCB2*, and *TAS2R13* in FuP. (B) The *TAS2Rs* located on chromosome 11 (*TAS2R9* and *TAS2R12-25*) appeared to be robustly expressed in subsets of TRCs, whereas only weak signals were observed for the *TAS2Rs* located on chromosomes 3 (*TAS2R1-8* and *TAS2R10-11*) and 6 (*TAS2R26*). *Tas2Rs* are arranged according to the locations on the chromosomes (see Figure S1). n = 2 (numbers of sections ≥4) for *TAS2R1-8*, *10*-*11*, *21*, *23*, and *26*, n = 1 (numbers of sections ≥2) for *TAS2R9*, *12, 14*-*20*, *22*, and *24*-*25*. Scale bars: 50 µm.

## Materials and Methods

### Macaques

This study was carried out in strict accordance with recommendations in the Guide for Care and Use of Nonhuman Primates of the Primate Research Institute, Kyoto University (Version 3, issued in 2010). This guideline was prepared based on the provisions of the Guidelines for Proper Conduct of Animal Experiments (June 1, 2006; Science Council of Japan), Basic Policies for the Conduct of Animal Experiments in Research Institutions under the Jurisdiction of the Ministry of Health, Labor and Welfare (effective on June 1, 2006; Ministry of Health, Labor and Welfare (MHLW)), Fundamental Guidelines for Proper Conduct of Animal Experiment and Related Activities in Academic Research Institutions (Notice No. 71 of the Ministry of Education, Culture, Sports, Science and Technology (MEXT) dated June 1, 2006), and Standards Relating to the Care and Management of Laboratory Animals and Relief of Pain (Notice No. 88 of the Ministry of the Environment dated April 28, 2006). All of the animal experiments were approved by the Animal Ethics Committee of the Primate Research Institute, Kyoto University (Permit Numbers: 2010-C-24 and 2011-B-17). Briefly, animals were kept in cages with sufficient space (780 mm wide, 650 mm depth, and 800 mm height) in the air conditioned room with sufficient environmental enrichment. The animals were housed in 12-hour light-dark cycle conditions with a daytime light intensity of 150–300 lux and their intake of water, food, or selected nutrients was not restricted. In addition to normal pellet foods, the animals were occasionally fed sweet potatoes, fruits, and vegetables for nutrimental enrichment. To ameliorate suffering, anesthesia was induced by intramuscular injection of ketamine (2.5 mg/kg) with medetomidine (0.1 mg/kg) into the femoral or brachial muscle of the animals at the initial step of the experiments. After the animals were anesthetized and immobilized, pentobarbital sodium (25 mg/kg) was infused intravenously on the autopsy table. After the animals were deeply anesthetized, which was confirmed by the absence of pain response, they were sacrificed by bloodletting from the jugular vein. After a sufficient amount of time had elapsed, respiratory arrest, cardiac arrest, and pupillary dilatation were confirmed. Then, taste tissues from five rhesus macaques (*Macaca mulatta*; approximately 3-year-old males), which were scheduled for euthanasia not only for this study but also for other experimental purposes, were collected and embedded in O.C.T. compound (Sakura Finetek, Tokyo, Japan) by ourselves. All the experiments described above were performed at Primate Research Institute, Kyoto University.

**Figure 2:**
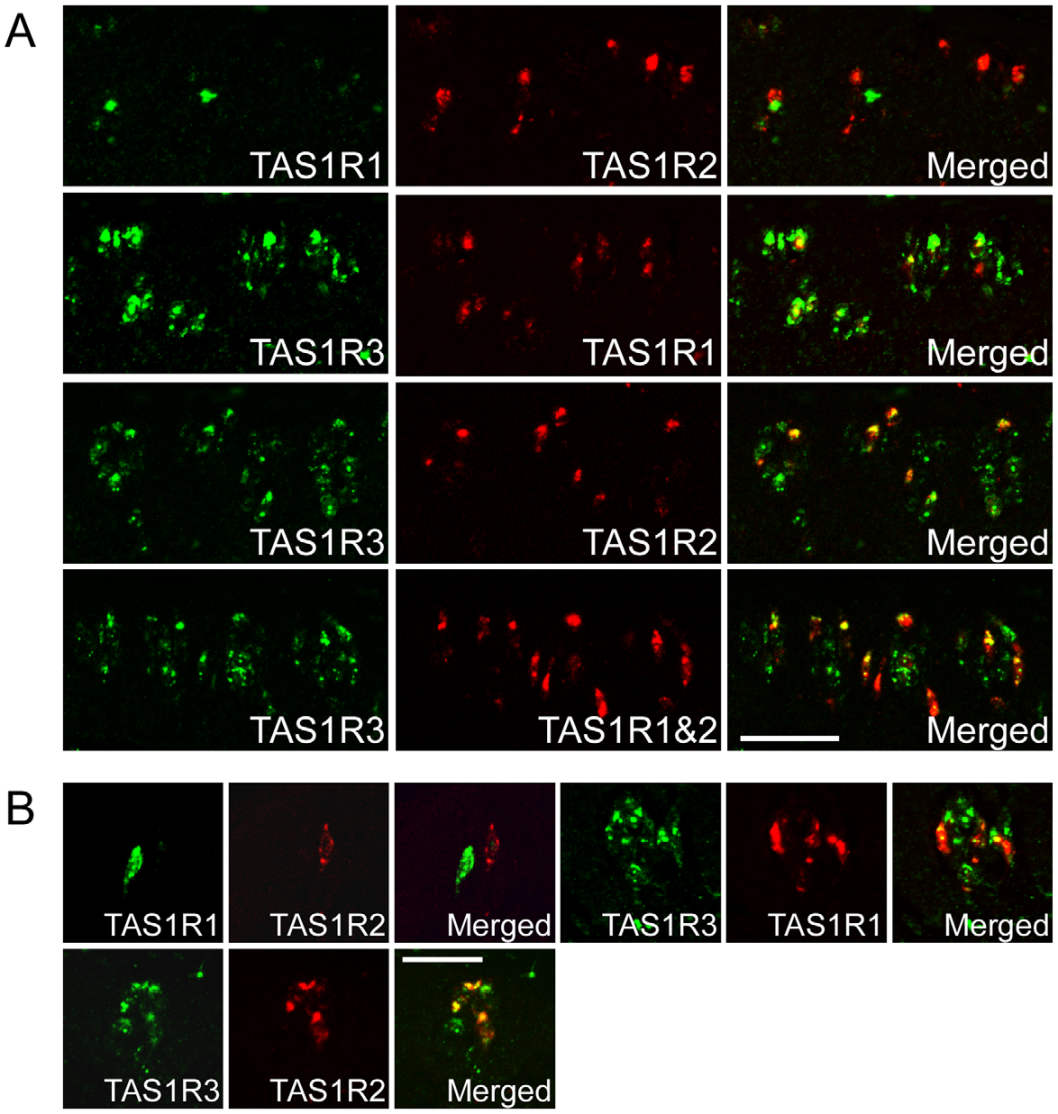
The co-expression relationships among three *TAS1Rs*. (A) *TAS1R1* and *TAS1R2* were exclusively expressed in different subsets of the *TAS1R3*-positive TRCs in the CvP. *In situ* hybridization using a mixed probe for *TAS1R1* and *TAS1R2* combined with a probe for *TAS1R3* revealed the presence of TRCs expressing *TAS1R3* alone. n = 2 (numbers of sections ≥4). (B) *TAS1R1* and *TAS1R2* were exclusively expressed in different subsets of the *TAS1R3*-positive TRCs in the FuP. The *TAS1R3*-positive TRCs in the FuP and CvP were classified into three types of cells: cells expressing *TAS1R1*+*TAS1R3*, those expressing *TAS1R2*+*TAS1R3*, and those expressing *TAS1R3* alone. n = 1 or 2 (numbers of sections ≥10). Scale bars: 50 µm.

**Figure 3:**
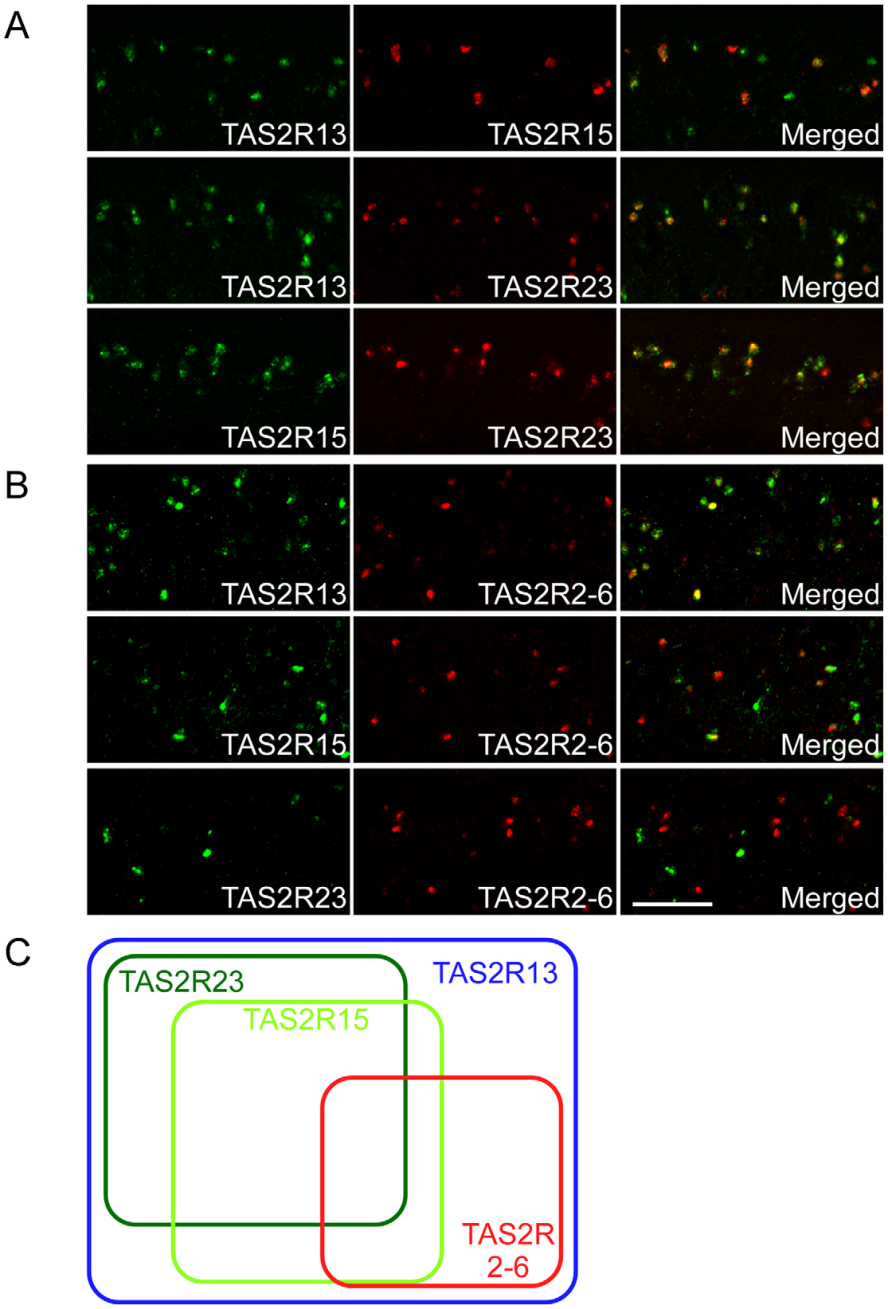
The co-expression relationships among *TAS2Rs*. (A) *TAS2R13*, *TAS2R15*, and *TAS2R23* reside in different *TAS2R* gene clusters on chromosome 11. Almost all of the *TAS2R15*- and *TAS2R23*-positive TRCs were also positive for *TAS2R13*, whereas *TAS2R15*-positive TRCs partially overlapped with those expressing *TAS2R23*. n = 1 or 2 (numbers of sections ≥2). (B) When we compared the TRCs expressing *TAS2Rs* located on different chromosomes, almost all of the TRCs labeled with a mixed probe for *TAS2R2-6* were also positive for *TAS2R13*, but they partially overlapped with the TRCs expressing *TAS2R15* or *TAS2R23*. n = 1 or 2 (numbers of sections ≥2). (C) A Venn diagram illustrating the co-expression relationships among the *TAS2Rs*. Each TRC sensing bitter compounds expresses various combinations of *TAS2Rs*. Scale bar: 50 µm.

**Figure 4:**
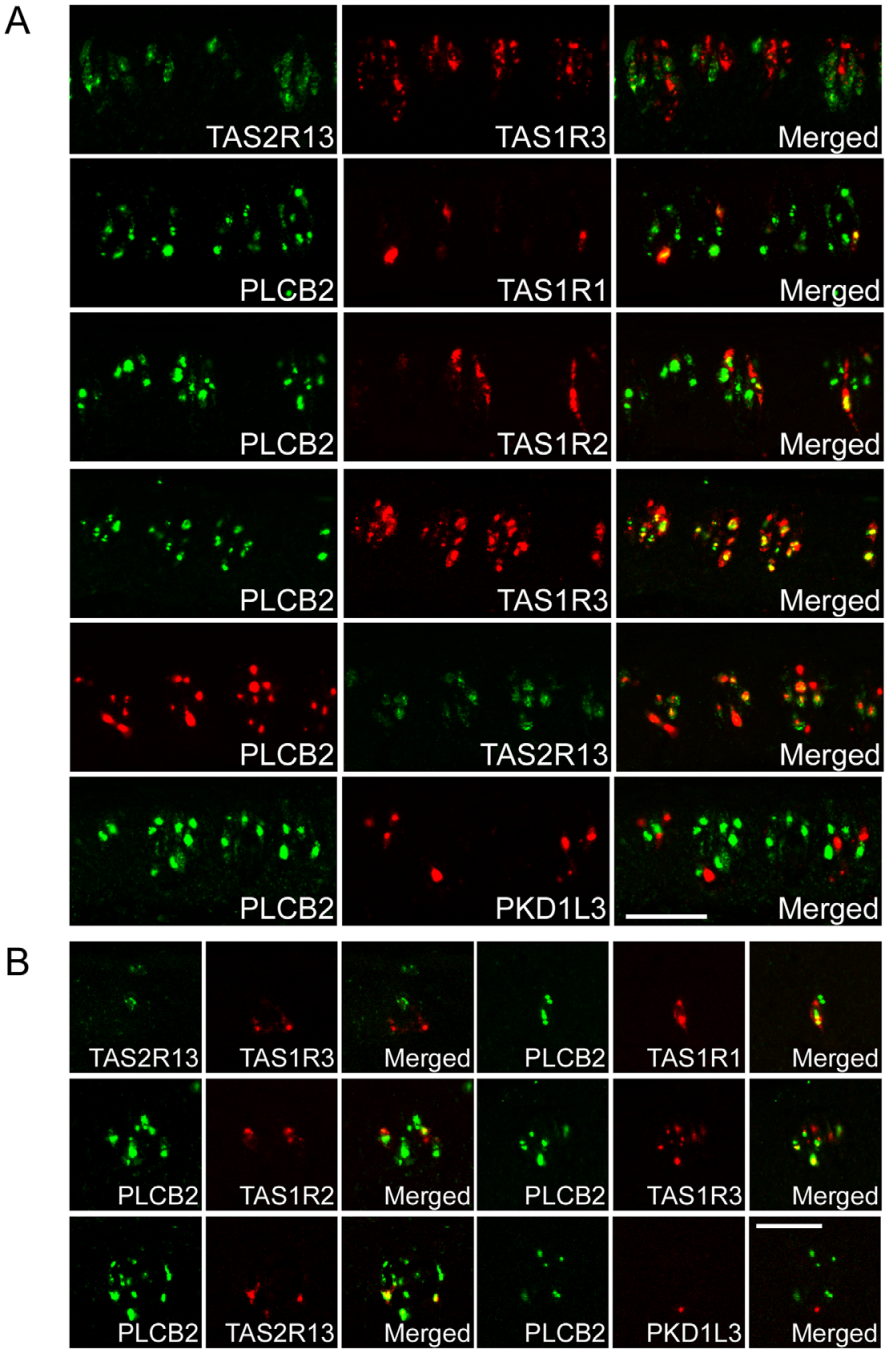
The co-expression relationships among taste receptors. (A) In the CvP, the *TAS1R3*-positive TRCs were negative for *TAS2R13*. The *PLCB2*-positive TRCs, which include *TAS1R1*-, *TAS1R2*-, *TAS1R3*-, and *TAS2R13*-positive TRCs, were negative for *PKD1L3*. n = 1 (numbers of sections ≥2). (B) In the FuP, the *TAS1R3*-positive TRCs were negative for *TAS2R13*. The *PLCB2*-positive TRCs, which include *TAS1R1*-, *TAS1R2*-, *TAS1R3*-, and *TAS2R13*-positive TRCs, were negative for *PKD1L3*. n = 1 or 2 (numbers of sections ≥10). Scale bars: 50 µm.

**Figure 5:**
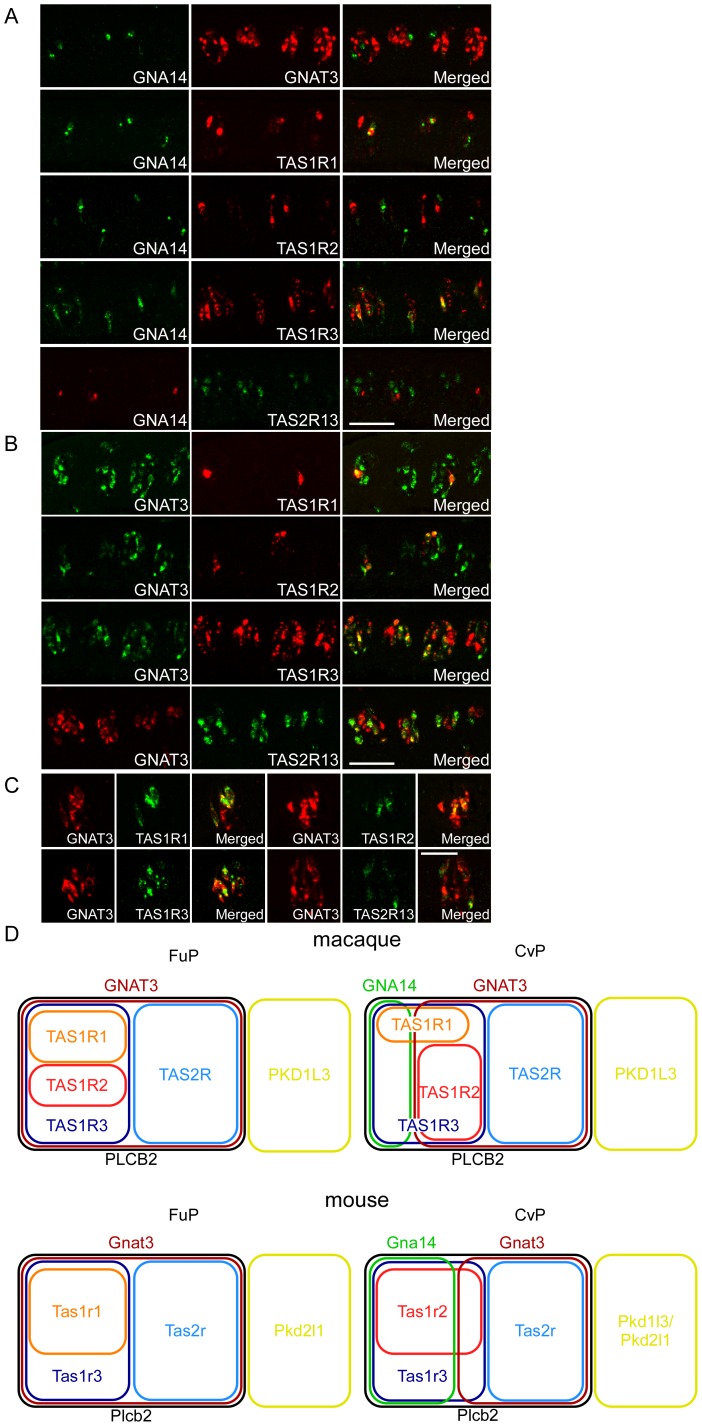
The co-expression relationships among taste receptors and G protein α subunits. (A) In the CvP, *GNA14* was expressed in a much smaller population of TRCs than *GNAT3* and in a mutually exclusive manner. The *GNA14*–positive TRCs were distinct from those expressing *TAS1R2* and *TAS2R13*, but they were subsets of the *TAS1R3*-positive TRCs and partially overlapped with the *TAS1R1*-positive TRCs. n ≥1 (numbers of sections ≥2). (B) In the CvP, *TAS1R2* and *TAS2R13* were expressed in subsets of the *GNAT3*–positive TRCs, which partially overlapped with the *TAS1R1*- and *TAS1R3*-positive TRCs. n ≥2 (numbers of sections ≥4). (C) In the FuP, *TAS1R1*, *TAS1R2*, *TAS1R3*, and *TAS2R13* were expressed in subsets of *GNAT3*-positive TRCs. n ≥1 (numbers of sections ≥10). (D) Venn diagram illustrating the co-expression relationships among taste receptors and signal transduction molecules in the macaque and the mouse. Scale bars: 50 µm.

### Database Search and Cloning

The TBLASTN program was used to search for genomic sequences showing significant identity with human *TAS1Rs*, *TAS2Rs*, *GNAT3*, *GNA14*, and *PLCB2* in the public genome database of the rhesus macaque (http://www.ensembl.org/Macaca_mulatta/Info/Index). The macaque *TAS2Rs* were named following the nomenclature proposed by Dong *et al* [28]. The cDNA sequence corresponding to G756-E852 of T1R3, which was unknown due to the lack of a genomic sequence, was obtained by 3′-RACE using 3′-Full RACE Core Set (Takara Bio Inc., Shiga, Japan). The entire coding regions of *TAS1R1*, *TAS1R2*, *TAS1R3*, *TAS2Rs*, *GNAT3*, and *GNA14* and partial coding regions of *PKD1L3* (C42-Y749) and *PLCB2* (M1-K192), which were amplified from macaque cDNA synthesized from epithelial tissues containing circumvallate papillae or genomic DNA extracted from tongue tissue, were used as probes.

### *In Situ* Hybridization (ISH)

Fresh frozen sections of tongue, 10 µm thick, were placed on MAS-coated glass slides (Matsunami Glass, Kishiwada, Japan). For ISH, the sections were fixed with 4% paraformaldehyde (PFA) in phosphate-buffered saline (PBS) and treated with proteinase K (6.4 µg/ml for 5 min) followed by acetylation. Prehybridization (at 58°C for 1 hour), hybridization (at 58°C, 2 O/N), washing (0.2 x SSC at 58°C), and development (NBT-BCIP) were performed using digoxigenin-labeled probes as described previously [17]. Double-label fluorescence ISH was performed with digoxigenin- and fluorescein-labeled RNA probes as described previously [29]. In brief, the probes were detected by incubation with a peroxidase-conjugated anti-digoxigenin antibody and a peroxidase-conjugated anti-fluorescein antibody (Roche, Indianapolis, IN, USA), followed by incubation with TSA-AlexaFluor 555 and TSA-AlexaFluor 488 (Invitrogen, Carlsbad, CA, USA) using the tyramide signal amplification method. Stained images were obtained using a fluorescence microscope (BX51; Olympus, Tokyo, Japan) equipped with a cooled CCD digital camera (DP71; Olympus) or a confocal laser-scanning microscope (FV500; Olympus).

## Results and Discussion

### Expression of Taste Receptors and Signal Transduction Molecules in the Fungiform and Circumvallate Papillae

To examine the tissue distributions of expression of genes involved in taste signal transduction, we conducted *in situ* hybridization on sections of the FuP and CvP using the following genes as probes: *TAS1R1*, *TAS1R2*, *TAS1R3*, *TAS2Rs*, *PKD1L3*, *GNAT3*, *GNA14*, and *PLCB2*. In the CvP, three *TAS1Rs*, *PKD1L3*, *GNAT3*, *GNA14*, and *PLCB2* were robustly expressed in subsets of TRCs (Figure 1A). Certain *TAS2Rs*, such as *TAS2R13*, were robustly expressed in subsets of TRCs, whereas only weak signals were observed for other *TAS2Rs*, including those located on chromosomes 3 and 6 (Figures 1A and B; Figure S1). In the FuP, three *TAS1Rs*, *GNAT3*, and *PLCB2*, but not *GNA14*, *w*ere robustly expressed in subsets of TRCs, whereas *TAS2R13* and *PKD1L3* were weakly expressed in subsets of TRCs (Figure 1A). It should be noted that *TAS1R1*, *TAS1R2*, and *TAS2R13*, as well as *PKD1L3* [25], were expressed in both the FuP and the CvP.

### Co-expression Relationships among Taste Signal Transduction Molecules

To compare the TRCs expressing each taste signal transduction molecule, we next performed double-label fluorescence ISH. T1R1 and T1R2 form heteromers with T1R3 to function as umami (savory) and sweet taste receptors, respectively [4], [5], [30]. *TAS1R1* and *TAS1R2* were exclusively expressed in different subsets of *TAS1R3*-positive TRCs in the FuP and CvP (Figure 2; Tables S1 and S2). In the CvP, approximately 20% and 40% of *TAS1R3*-positive TRCs were also positive for *TAS1R1* and *TAS1R2*, respectively. Experiments using a mixed probe for *TAS1R1* and *TAS1R2* combined with a probe for *TAS1R3* confirmed that approximately 40% of *TAS1R3*-positive TRCs were negative for both *TAS1R1* and *TAS1R2* (Figure 2A and data not shown). In the FuP, approximately 40% and 30% of *TAS1R3*-positive TRCs were also positive for *TAS1R1* and *TAS1R2*, respectively (Figure 2B; Table S2). In summary, *TAS1R3*-positive TRCs in the FuP and CvP can be classified into three types of cells: cells expressing *TAS1R1*+*TAS1R3*, those expressing *TAS1R2*+*TAS1R3*, and those expressing *TAS1R3* alone.

A previous gene expression analysis using microarrays in the taste buds of the cynomolgus macaque collected by laser capture microdissection revealed that *TAS1R1* and *TAS1R2* were more highly expressed in the taste buds of the FuP than in those of the CvP [24]. Our ISH analysis quantified the expression of the genes involved in taste signal transduction at the cellular mRNA level. Consequently, the majority of genes, including *TAS1R1* and *TAS1R2*, showed more uniform expression patterns in the FuP and the CvP of macaques than in those of rodents. These results are consistent with previous findings from gustatory nerve recordings in the rhesus macaque showing that both the chorda tympani and glossopharyngeal nerves, which innervate the FuP and CvP, respectively, responded to a variety of basic taste compounds [31].

*TAS2R13*, *TAS2R15*, and *TAS2R23*, which were robustly expressed in subsets of the TRCs in the CvP (Figure 1B), reside in different *TAS2R* gene clusters on chromosome 11 [28] (Figure S1; http://www.ensembl.org/Macaca_mulatta/Info/Index). Almost all the *TAS2R15*- and *TAS2R23*-positive TRCs were also positive for *TAS2R13*, whereas *TAS2R15*-positive TRCs partially overlapped with those expressing *TAS2R23* (Figure 3A; Table S3). We used a mixed probe for *TAS2R2*, *TAS2R3*, *TAS2R4*, *TAS2R5*, and *TAS2R6* because only weak signals were detected for each *TAS2R* located on chromosome 3 (Figure 1B). When we compared the TRCs expressing *TAS2Rs* located on different chromosomes, almost all the TRCs labeled with the mixed probe were also positive for *TAS2R13*, but they partially overlapped with TRCs expressing *TAS2R15* or *TAS2R23* (Figure 3B; Table S3). These results demonstrate that each TRC sensing bitter compounds expresses various combinations of *TAS2Rs* (Figure 3C), as in the case of human *TAS2Rs* [23].

We next compared the TRCs expressing taste receptors for different basic taste modalities. We chose *TAS2R13* as a representative *TAS2R* because almost all the TRCs expressing other *TAS2Rs* that we tested were included in the *TAS2R13*-positive TRCs, as described above (Figure 3; Table S3). *TAS1R1* and *TAS1R2* were exclusively expressed in different subsets of *TAS1R3*-positive TRCs (Figure 2). *TAS1R3*-positive TRCs were negative for *TAS2R13* in the CvP (Figure 4A) and in the FuP (Figure 4B). *TAS1R1*-, *TAS1R2*-, *TAS1R3*-, and *TAS2R13*-positive TRCs were also positive for *PLCB2* in the CvP (Figure 4A; Table S1) and in the FuP (Figure 4B), as in the case of other vertebrates such as rodents and fish [29], [32]. *PLCB2*-positive TRCs were negative for *PKD1L3* in the CvP (Figure 4A) and in the FuP (Figure 4B). In summary, *TAS1R1*, *TAS1R2*, *TAS2Rs*, and *PKD1L3* were exclusively expressed in different subsets of the TRCs in the FuP and CvP (Figure 5D). Thus, these results suggest that the TRCs detecting different basic taste modalities are mutually segregated in macaque taste buds.

Finally, we focused on two genes encoding G protein α subunits, *GNAT3* and *GNA14*, which are specifically expressed in subsets of rodent TRCs [10], [13], [22], [33]. In the CvP, *GNA14* was expressed in a much smaller population of TRCs than *GNAT3* and in a mutually exclusive manner (Figures 1A and 5A; Table S1). *GNA14*–positive TRCs were distinct from those expressing *TAS1R2* and *TAS2R13*, but they formed subsets of *TAS1R3*-positive TRCs and partially overlapped with *TAS1R1*-positive TRCs (Figure 5A; Table S1). In contrast, *TAS1R2* and *TAS2R13* were expressed in different subsets of *GNAT3*–positive TRCs, which partially overlapped with *TAS1R1*- and *TAS1R3*-positive TRCs (Figure 5B; Table S1). It should be noted that *TAS1R2* was co-expressed with *GNAT3* but not with *GNA14* in macaques, whereas *Tas1r2* was primarily co-expressed with *Gna14* but not with *Gnat3* in rodents [10], [13], [22]. In the FuP, *GNAT3*, but not *GNA14*, was expressed in TRCs (Figure 1A). *TAS1R1*, *TAS1R2*, *TAS1R3*, and *TAS2R13* were expressed in subsets of *GNAT3*-positive TRCs (Figure 5C; Table S2). These results suggest that *GNAT3* plays a pivotal role in mediating sweet, bitter, and umami tastes in macaques.

### Conclusions

We investigated the expression of taste receptors and signal transduction molecules in the FuP and CvP of the rhesus macaque and further examined the co-expression relationships among these genes. The majority of genes exhibited more uniform expression patterns in the macaque FuP and CvP than in these papillae in rodents (Figure 5D). Intriguingly, there were several differences between the expression profiles of macaques and rodents. First, *TAS1R1* and *TAS1R2* were more uniformly expressed in both the FuP and the CvP of macaques than in rodents. Second, *TAS1R2* was co-expressed with *GNAT3* in the CvP but not with *GNA14*. Third, macaque *TAS2Rs* were expressed in heterogeneous populations of TRCs in the CvP. These results suggest that the molecular mechanisms underlying taste transduction in primates, including humans, may be different from those in rodents and that the macaque is an important model organism for taste perception in humans.

## Supporting Information

Figure S1 Schematic drawing illustrating the locations of macaque *TAS2Rs* on the chromosomes. *TAS2R1-8*, *TAS2R11*, and *TAS2R38* are located on chromosome 3, whereas only *TAS2R26* is located on chromosome 6. The other 15 *TAS2Rs* are located on chromosome 11, although precise location of *TAS2R9* has not been determined. *TAS2R13*, *TAS2R15*, and *TAS2R23* reside in different *TAS2R* gene clusters on the chromosome 11.
(TIF)

Table S1 The percentages of *TAS1Rs*, *GNAT3*, *GNA14*, and *PLCB2* co-expression in the circumvallate taste buds. The percentage values were calculated by dividing the number of cells expressing both gene X and gene Y by the number of cells expressing gene X.
(DOCX)

Table S2 The percentages of *TAS1Rs*, *TAS2R13*, and *GNAT3* co-expression in the fungiform taste buds. The percentage values were calculated by dividing the number of cells expressing both gene X and gene Y by the number of cells expressing gene X.
(DOCX)

Table S3 The percentages of *TAS2R* co-expression in the circumvallate taste buds. The percentage values were calculated by dividing the number of cells expressing both gene X and gene Y by the number of cells expressing gene X.
(DOCX)
