# Supplementary figures and images for: Expression Analysis of Taste Signal Transduction Molecules in the Fungiform and Circumvallate Papillae of the Rhesus Macaque, Macaca mulatta
Source: PLoS One. 2012 Sep 21;7(9):e45426. doi: 10.1371/journal.pone.0045426 (PMC3448732; doi:10.1371/journal.pone.0045426)

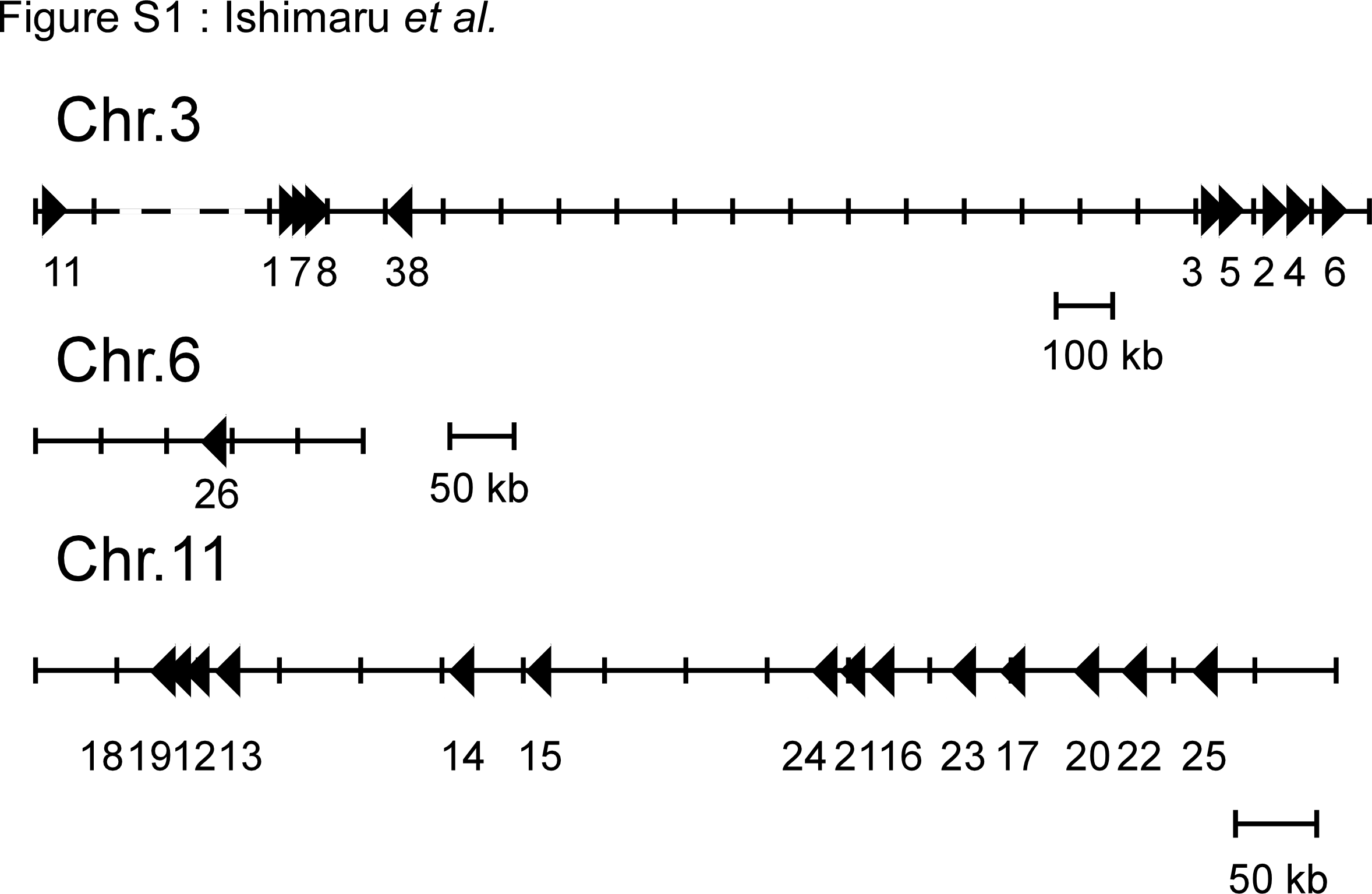

Supplement: Figure S1 — Schematic drawing illustrating the locations of macaque TAS2Rs on the chromosomes. TAS2R1-8, TAS2R11, and TAS2R38 are located on chromosome 3, whereas only TAS2R26 is located on chromosome 6. The other 15 TAS2Rs are located on chromosome 11, although precise location of TAS2R9 has not been determined. TAS2R13, TAS2R15, and TAS2R23 reside in different TAS2R gene clusters on the chromosome 11. (TIF) [file pone.0045426.s001.tif]
