# Supplementary material for: Expression Analysis of Taste Signal Transduction Molecules in the Fungiform and Circumvallate Papillae of the Rhesus Macaque, Macaca mulatta
Source: PLoS One. 2012 Sep 21;7(9):e45426. doi: 10.1371/journal.pone.0045426 (PMC3448732; doi:10.1371/journal.pone.0045426)
Supplement: Table S1 — The percentages of TAS1Rs, GNAT3, GNA14, and PLCB2 co-expression in the circumvallate taste buds. The percentage values were calculated by dividing the number of cells expressing both gene X and gene Y by the number of cells expressing gene X. (DOCX) [file pone.0045426.s002.docx]

Table S1. The percentages of *TAS1Rs*, *GNAT3*, *GNA14*, and *PLCB2* co-expression in the circumvallate taste buds

| X Y | TAS1R1 | TAS1R2 | TAS1R3 | GNAT3 | GNA14 | PLCB2 |
| --- | --- | --- | --- | --- | --- | --- |
| TAS1R1 |  | 0%  (0/48) | 18% (32/176) | 12% (30/250) | 40% (23/57) | 17% (23/138) |
| TAS1R2 | 0%  (0/14) |  | 37% (100/273) | 30% (68/228) | 8%  (9/108) | 25% (33/133) |
| TAS1R3 | 100% (32/32) | 100% (100/100) |  | 82% (125/153) | 100% (47/47) | 80% (86/107) |
| GNAT3 | 68% (30/44) | 92% (68/74) | 72% (125/174) |  | 0%  (0/9) | 66% (61/92) |
| GNA14 | 46% (23/50) | 7%  (9/132) | 26% (47/182) | 0%  (0/39) |  | 14% (18/133) |
| PLCB2 | 100% (23/23) | 87% (33/38) | 84% (86/103) | 91% (61/67) | 100% (18/18) |  |

The percentage values were calculated by dividing the number of cells expressing both gene X and gene Y by the number of cells expressing gene X.
