# Supplementary material for: Expression Analysis of Taste Signal Transduction Molecules in the Fungiform and Circumvallate Papillae of the Rhesus Macaque, Macaca mulatta
Source: PLoS One. 2012 Sep 21;7(9):e45426. doi: 10.1371/journal.pone.0045426 (PMC3448732; doi:10.1371/journal.pone.0045426)
Supplement: Table S2 — The percentages of TAS1Rs, TAS2R13, and GNAT3 co-expression in the fungiform taste buds. The percentage values were calculated by dividing the number of cells expressing both gene X and gene Y by the number of cells expressing gene X. (DOCX) [file pone.0045426.s003.docx]

Table S2. The percentages of *TAS1Rs*, *TAS2R13*, and *GNAT3* co-expression in the fungiform taste buds

| X Y | TAS1R1 | TAS1R2 | TAS1R3 | TAS2R13 | GNAT3 |
| --- | --- | --- | --- | --- | --- |
| TAS1R1 |  | 5%  (1/19) | 44%  (88/202) |  | 40% (81/203) |
| TAS1R2 | 5%  (1/19) |  | 31%  (75/240) |  | 35% (75/216) |
| TAS1R3 | 96%  (88/92) | 97%  (75/77) |  | 0%  (0/23) | 76%  (53/70) |
| TAS2R13 |  |  | 0%  (0/9) |  | 45%  (46/103) |
| GNAT3 | 84%  (81/96) | 100%  (75/75) | 100%  (53/53) | 100%  (46/46) |  |

The percentage values were calculated by dividing the number of cells expressing both gene X and gene Y by the number of cells expressing gene X.
