# Supplementary material for: Expression Analysis of Taste Signal Transduction Molecules in the Fungiform and Circumvallate Papillae of the Rhesus Macaque, Macaca mulatta
Source: PLoS One. 2012 Sep 21;7(9):e45426. doi: 10.1371/journal.pone.0045426 (PMC3448732; doi:10.1371/journal.pone.0045426)
Supplement: Table S3 — The percentages of TAS2R co-expression in the circumvallate taste buds. The percentage values were calculated by dividing the number of cells expressing both gene X and gene Y by the number of cells expressing gene X. (DOCX) [file pone.0045426.s004.docx]

Table S3. The percentages of *TAS2R* co-expression in the circumvallate taste buds

| X Y | TAS2R13 | TAS2R15 | TAS2R23 | TAS2R2-6 |
| --- | --- | --- | --- | --- |
| TAS2R13 |  | 98%  (51/52) | 100%  (82/82) | 93%  (70/75) |
| TAS2R15 | 42%  (51/122) |  | 81%  (68/84) | 50%  (31/62) |
| TAS2R23 | 55%  (82/155) | 86%  (68/79) |  | 9%  (6/67) |
| TAS2R2-6 | 31%  (70/229) | 30%  (31/105) | 8%  (6/72) |  |

The percentage values were calculated by dividing the number of cells expressing both gene X and gene Y by the number of cells expressing gene X.
